# Retraction Note: DNA methylome profiling at single-base resolution through bisulfite sequencing of 5mC-immunoprecipitated DNA

**DOI:** 10.1186/s12896-020-00619-w

**Published:** 2020-05-11

**Authors:** Zhen Jia, Yueyi Shi, Lei Zhang, Yipeng Ren, Tong Wang, Lejun Xing, Baorong Zhang, Guolan Gao, Rongfa Bu

**Affiliations:** 1 Department of Obstetrics and Gynecology, Beijing Aviation General Hospital, Beijing, 100012 China; 2 Department of Stomatology, Chinese PLA General Hospital, Beijing, 100853 People’s Republic of China; 3 Medical School, Nankai University, No.94 Weijin Road, Tianjin, 300070 China; 4 Department of Stomatology, Beijing Aviation General Hospital, Beijing, 100012 People’s Republic of China

**Correction Note: BMC Biotechnology (2018) 18:7**

**https://doi.org/10.1186/s12896-017-0409-7**

The Editor has retracted this article [1] due to substantial similarities with an unpublished manuscript by Yan Wang, Guanyu Ji, Jinyu Wu, Mingzhi Ye, Lee M. Butcher, Ning Li, Wanshi Cai, Xu Han, Wei Dai, Qi Liu, Zhimei Yuan, Xiuqing Zhang, and Zhong Sheng Sun. The authors have not responded to correspondence about this retraction.
